# MoO_2_ nanosheets embedded in amorphous carbon matrix for sodium-ion batteries

**DOI:** 10.1098/rsos.170892

**Published:** 2017-10-18

**Authors:** Hong He, Yuhong Man, Jingang Yang, Jiale Xie, Maowen Xu

**Affiliations:** 1 Faculty of Materials and Energy, Southwest University, Chongqing 400715, People's Republic of China; 2 Chongqing Key Laboratory for Advanced Materials & Technologies of Clean Electrical Power Sources, Chongqing 400715, People's Republic of China; 3 Institute of Materials Science and Devices, Suzhou University of Science and Technology, Suzhou 215011, People's Republic of China

**Keywords:** MoO_2_ nanosheets, sodium-ion batteries, hydrothermal method, cycle performance

## Abstract

MoO_2_ nanosheets embedded in the amorphous carbon matrix (MoO_2_/C) are successfully synthesized via a facile hydrothermal method and investigated as an anode for sodium-ion batteries. Because of the efficient ion transport channels and good volume change accommodation, MoO_2_/C delivers a discharge/charge capacity of 367.8/367.0 mAh g^−1^ with high coulombic efficiency (99.4%) after 100 cycles at a current density of 50 mA g^−1^.

## 1. Introduction

Currently, the high demand for clean and renewable energy has fuelled the exploration of advanced storage systems. Because of the low cost and abundant supply of sodium, room temperature rechargeable sodium-ion batteries (SIBs) have attracted much attention as a potential alternative device for lithium ion batteries [1–3]. Unfortunately, the large ion radius of sodium (0.102 nm) makes it difficult to find suitable electrode materials to realize reversible insertion/extraction of sodium ions, and it has become a great challenge to design and fabricate suitable electrode materials for novel SIBs [4–6].

Recently, carbonaceous-based anode materials have undergone great development for non-aqueous SIBs, such as graphite, hard carbon and meso-carbon microbeads [7–9]. However, these materials present a relatively low capacity of less than 300 mAh g^−1^. To obtain high capacity, several other types of materials have been explored, such as metals and alloys (Sn, Sb and SnSb) [10,11] and metal chalcogenides (TiS_2_, Sb_2_S_3_, FeS_2_, MoS_2_ and so on) [12–14]. In the continued search for anodes of SIBs, transition metal oxides, with superior sodium storage properties, are of great interest because of their potential to deliver high and stable specific capacities [15–17]. For example, Balaya investigated the sodium storage of Fe_3_O_4_ anodes, which exhibited an initial discharge capacity of 643 mAh g^−1^ but was accompanied with nearly 50% irreversible capacity and poor capacity retention [18,19]. α-MoO_3_ anodes showed first sodiation and de-sodiation capacities of 771 and 410 mAh g^−1^, respectively [20,21].

Molybdenum oxides, including MoO_3_ and MoO_2_, have been a new family of anode materials for SIBs. Molybdenum oxides as electrode materials possess some superior advantages, such as low electrical resistivity, high theoretical specific capacity of approximately 840 mAh g^−1^ (MoO_2_, an insertion of four sodiums), high thermal and chemical stability, and affordable cost [4,22,23]. MoO_2_ and its carbonaceous composites have been broadly investigated in lithium ion batteries. MoO_2_ microcapsules exhibit a high specific capacity of 749.3 mA h g^−1^ in the first discharge at a rate of 1 C and high reversible capacity of 623.8 mAh g^−1^ after 50 cycles [24]. The MoO_2_/multiwalled carbon nanotubes (MWCNT) hybrid shows an enhanced reversible lithium storage capacity (1143 mA h g^−1^ at a current density of 100 mA g^−1^ after 200 cycles), high rate capability (408 mA h g^−1^ at a high C-rate of 1000 mA g^−1^) and good cycling stability [25].

To date, there are a few reports on MoO_2_-based composites for SIBs. Huang *et al*. reported that MoO_2_ nanoparticles (approx. 100 nm) anchored on graphene oxide (MoO_2_/GO) shows a discharge gravimetric capacity of 483 mAh g^−1^ at the current density of 100 mA g^−1^ after 10 cycles [26]. However, GO is a wide band gap semiconductor of approximately 3.5 eV. This indicates its low conductivity for charge transfer and transport, which is not beneficial for high capacity and high power density. Therefore, it is expected that another type of carbon with high conductivity can be used to enhance the specific capacity and the power density. Moreover, MoO_2_@C nanoflowers synthesized by a grinding method deliver a reversible capacity of 172 mAh g^−1^ at 0.1 A g^−1^ for SIBs [27]. The low capacity may come from the imperfect coating of carbon by the grinding method. For SIBs, a poor capacity retention of MoO_2_ is one of critical issues for long-term cycling, which is mainly attributed to the large volume variation during Na^+^ insertion/extraction processes and the agglomeration of MoO_2_ particles. As above, combining the design of MoO_2_ nanostructures and coating the MoO_2_ with conductive carbon may be an effective method to boost the sodium storage performances of MoO_2_.

Herein, MoO_2_ nanosheets embedded in the amorphous carbon matrix (MoO_2_/C nanosheets) were successfully synthesized via a facile hydrothermal method and investigated as anode materials for SIBs. MoO_2_/C nanosheets display a stable cycling performance and high rate capability, and a reversible capacity of 367.8 mAh g^−1^ can be achieved at 50 mA g^−1^ over 100 cycles. The improved mechanism and some scientific insights are also discussed.

## 2. Material and methods

### 2.1. Synthesis of MoO_2_/C nanosheets

Molybdenic acid was dispersed in deionized water with continuous stirring. t-dodecanethiol was introduced into this suspension and this was agitated by sonication for 30 min. After that, the mixture was transferred to an autoclave and the temperature raised to 200°C for 12 h. The resulting dark precipitate was impregnated with glucose solution by stirring overnight. The impregnated material was then collected, dried and finally heated at 400°C for 1 h under Ar atmosphere.

### 2.2. Characteristics of materials

Scanning electron microscope (SEM) and transmission electron microscope (TEM) testing were performed on a JSM-6610 and a JEM-2010 electron microscope, respectively. X ray diffraction (XRD) measurements were carried out on a XRD-7000 X-ray diffractometer using Cu K*α* radiation at *λ* = 0.154 nm. The thermal stability was measured with a NETZSCH 409PC in dry air. The specific surface area and pore-size distribution were determined by the Brunauer–Emmett–Teller (BET) measurement by employing an ASAP-2010 surface area analyser. Raman spectra were collected by an Invia Refl (Renishaw, UK) under ambient conditions, from 2000 to 400 cm^–1^ with 532.8 nm laser light. X-ray photoelectron spectroscopy (XPS) characterization was carried out in an ESCALAB 250Xi electron spectrometer.

### 2.3. Electrochemical characterizations

Anodes were prepared on Cu foils with slurries consisting of 80 wt.% active material, 10 wt.% polyvinylidenefluoride (PVDF) as a binder, 10 wt.% super P carbon as a conductive medium and Nmethyl-pyrrolidone (NMP) as a solvent. The electrodes were then dried at 80°C overnight in a vacuum oven. The coin-type cells (CR 2032) were assembled with sodium metal as the counter electrode. NaClO_4_ (1.0 M) in a mixture of ethylene carbonate(EC)/propylene carbonate (PC) (in a volume ratio of 1 : 1) was taken with a 5 wt% fluoroethylene carbonate (FEC) additive as the electrolyte, and the separator was a microporous membrane (Celgard 2135). The cells were assembled in an argon-filled glove box. The galvanostatic charge/discharge experiments were conducted at a voltage of 0.05–2.5 V versus Na^+^/Na on a land system (China). All the electrochemical tests were carried out at ambient temperature.

## 3. Results and discussion

**Figure 1.**
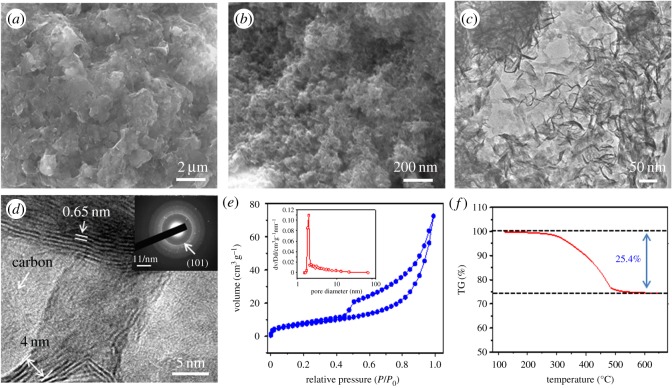
SEM images (*a*,*b*) and TEM images (*c*,*d*) of the MoO_2_/C nanosheets. N_2_ adsorption/desorption isotherms (*e*) and TGA results of the MoO_2_/C nanosheets (*f*).

**Figure 2.**
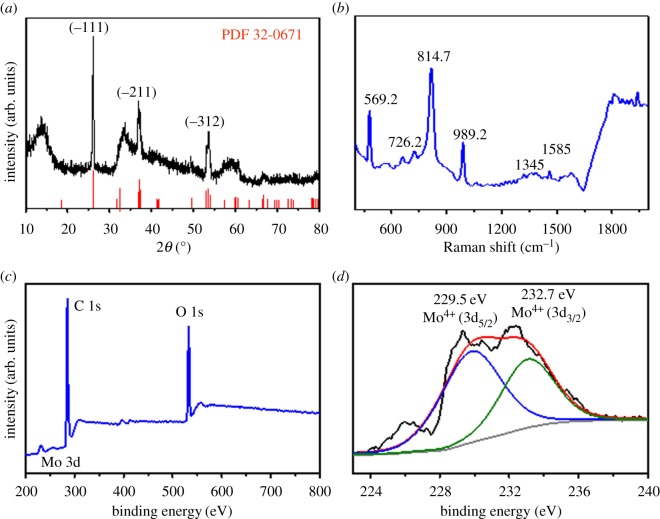
XRD patterns (*a*), Raman spectrum (*b*), XPS survey (*c*), and high-resolution XPS spectra for Mo 3d (*d*) of the MoO_2_/C nanosheets.

The synthesis of MoO_2_/C nanosheets involves interfacial self-assembly of laminar MoO_3_ nanosheets and thermally converted to MoO_2_/C by glucose through an impregnation reduction–carbonization process [28]. At the initial stage, the oil–water interface forms with the hydrophilic –SH ends arranging towards water. Then Mo ions can coordinate with –SH groups, and MoO_3_ nanosheets grow and stack together by self-assembly. Finally, the MoO_3_ nanosheets were thermally reduced to MoO_2,_ and MoO_2_/C could be obtained with the use of glucose. Figure 1*a–d* shows the SEM and TEM images of the as-prepared MoO_2_/C composite. It can be seen from figure 1*a*,*b* that the composites exhibited a wrinkled and corrugation structure. That is, the composite shows a two-dimensional nanosheet morphology. Furthermore, the TEM images (figure 1*c*,*d*) validate the presence of MoO_2_ nanosheets, which are embedded in the amorphous matrix. The amorphous phase in figure 1*c* could be attributed to carbon originated from the carbonization of glucose at a low temperature of 200°C [28]. The thickness of the MoO_2_ nanosheets composed of 6–12 layers is evaluated to be 4–8 nm. As in figure 1*d*, the interspace of the layer is around 0.65 nm. The large interspace suggests that the prepared MoO_2_/C is beneficial for rapid Na^+^ diffusion and intercalation. The selected area electron diffraction (SAED, inset in figure 1*d*) of the as-prepared composite presents the characteristics of polycrystalline rings and shows that it is the (101) plane of MoO_2_, corresponding to a d-spacing of 0.28 nm. The texture structure of the MoO_2_/C nanosheets was measured by the nitrogen absorption–desorption isotherms (figure 1*e*), showing a type IV isotherm with a surface area of 43.7 m^2^ g^−1^ and many mesopores. As shown in the inset of figure 2*d*, the Barrett–Joyner–Halenda (BJH) pore size distribution of MoO_2_/C shows a most probable pore size of 1.98 nm, which is much larger than the diameter of Na^+^ (0.2 nm). The carbon content of the MoO_2_/C measured by TGA (figure 1*f*) shows that there is about 25.4 wt.% weight loss in the MoO_2_/C composite from 120°C to 650°C, which can be attributed to the oxidation of carbon (C + O_2_ → CO_2_). That is, the loading of MoO_2_ in the MoO_2_/C composite is estimated to be 74.6 wt.%.

Figure 2*a* shows the XRD pattern of the MoO_2_/C nanosheets. All the main peaks in the XRD patterns can be readily indexed to MoO_2_ (JCPDS no. 32-0671). The strong peaks at 26.1°, 36.9° and 53.6° correspond to the (−111), (−211) and (−312) reflections of MoO_2_. The XRD pattern confirms that all the MoO_3_ has been converted into MoO_2_. The strong peak at approximately 2*θ* = 13.78° corresponds to the interlayer spacing of adjacent layers of MoO_2_ (0.65 nm). This is in good agreement with the result of TEM characterization in figure 1*d*. Furthermore, no peak related to carbon can be observed in figure 2*a*, suggesting its amorphous state. This coincides with the morphology observed in figure 1*d*.

The structure of the MoO_2_/C probed by Raman spectroscopy is shown in figure 2*b*. Character bands of molybdenum oxide owing to bond vibration modes are clearly confirmed at 989.2 (Mo=O), 814.7 (O-Mo-O), 726.2 (O2-Mo) and 569.2 cm^−1^ (O1-Mo). The additional bands at 1345 and 1585 cm^−1^ can be attributed to D and G bands of amorphous carbon, respectively [28]. XPS was conducted to further examine the chemical composition of the surface of the MoO_2_ nanosheets (figure 2*c*,*d*). Figure 2*c* shows that the materials contain Mo, C and O. The high-resolution XPS spectrum of the Mo 3d clearly demonstrates that the composite is MoO_2_ with Mo^4+^ (229.5 and 232.7 eV), which is in good agreement with the reported literature [23].

**Figure 3.**
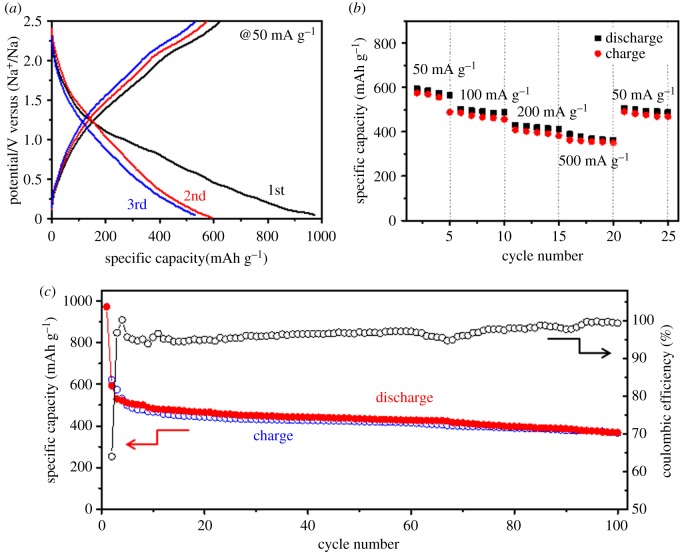
Charge/discharge curves (*a*), rate capability (*b*), and cycling performance (*c*) of MoO_2_/C nanosheets used as an anode for SIBs at 50 mA g^−1^.

The electrochemical performance of the MoO_2_/C nanosheets was investigated by using 2032 type Na cells. Figure 3*a* shows the discharge/charge profiles of MoO_2_/C at 50 mA g^−1^ between 0.05 and 2.5 V at the first three cycles. The initial discharge and charge capacities of MoO_2_/C are 971.9 mAh g^−1^ and 622.2 mAh g^−1^ with a coulombic efficiency of 64.0%. The specific capacity was calculated based on the weight of MoO_2_/C, because the contribution of super P carbon to the reversible capacity is negligible [6]. The capacity loss is mainly owing to the decomposition of electrolyte to form a solid–electrolyte interphase (SEI) film in the first cycle [29,30].

The rate capability of MoO_2_/C nanosheets is shown in figure 3*b*. It can be seen from figure 3*b* that the reversible capacities are 594, 502, 431, 389 and 505 mAh g^−1^ at constant current rates of 50, 100, 200, 500 and 50 mA g^−1^, respectively. The MoO_2_/C anode delivers a reversible capacity of 38 mAh g^−1^ at a current of 500 mA g^−1^. When the current rate returns to 50 mA g^−1^, 85.0% of the original capacity (505 mAh g^−1^) reverts, which means it has a high rate capability.

Figure 3*c* shows the cycling performance of MoO_2_/C nanosheets at 50 mA g^−1^ as an anode for sodium ion batteries. It is clearly seen that MoO_2_/C nanosheets had a stable cycling performance during the first 100 cycles, delivering a discharge/charge capacity of 367.8/367.0 mAh g^−1^ with high coulombic efficiency (99.4%) after the 100th cycle. The deviation of coulombic efficiency from 100% could be attributed to two factors: (i) the full reduction of MoO_2_ under a small current density makes it difficult for the complete extraction of Na^+^ in a consequent charging process; and (ii) the extensive discharge depth may bring about a collapse of the crystallographic structure of MoO_2_ [31]. By contrast, the discharge capacity of MoO_2_/graphene oxide nanoparticles reported by Huang *et al*. is only 345 mAh g^−1^ [26]. This demonstrates that amorphous carbon coated MoO_2_ has superior performance, which should be attributed to the better conductivity of amorphous carbon than that of graphene oxide.

The high rate capability and long-term cycling performance of MoO_2_/C are attributed to its unique features. That is, the ultrathin MoO_2_/C nanosheets can significantly decrease the stress/strain during the discharge/charge processes. MoO_2_ nanosheets which are embedded in the amorphous matrix can effectively accommodate the large volume change and prevent the aggregation of MoO_2_ nanosheets during the Na-ion insertion and extraction processes. This can maintain the mechanical integrity of the MoO_2_ electrode, and the nanosheets' structure serves as buffered spaces during the charge/discharge processes. Eventually, the nanosheets' structure can facilitate the mass transport between the electrolyte and electrode, reduce the sodium diffusion length, improve the electronic and ionic transport, and accommodate volume changes to enhance the electrochemical property of the composite electrode [21,28].

## 4. Conclusion

In summary, MoO_2_/C nanosheets were successfully synthesized via a facile hydrothermal method and investigated as anode materials for SIBs. Because of the efficient ion transport channels between the layers, the cycling performance of MoO_2_/C nanosheets as an anode for SIBs shows that the MoO_2_/C nanosheets exhibit a stable cycling performance and a high rate capability, delivering a discharge/charge capacity of 367.8/367.0 mAh g^−1^ with high coulombic efficiency (99.4%) after 100 cycles. The results reveal that the MoO_2_/C nanosheets are attractive candidates as anode materials for SIBs.

## Data accessibility

All primary data collected and analysed for this study have been deposited with Dryad and can be accessed at http://datadryad.org/resource/doi:10.5061/dryad.55678 [32].
